# Financial incentives to motivate treatment for hepatitis C with direct acting antivirals among Australian adults (The Methodical evaluation and Optimisation of Targeted IncentiVes for Accessing Treatment of Early-stage hepatitis C: MOTIVATE-C): protocol for a dose-response randomised controlled study

**DOI:** 10.1186/s13063-024-08212-8

**Published:** 2024-06-17

**Authors:** Parveen Fathima, Mark Jones, Reena D’Souza, James Totterdell, Nada Andric, Penelope Abbott, Richard Norman, Kirsten Howard, Wendy Cheng, Alisa Pedrana, Joseph S. Doyle, Jane Davies, Thomas Snelling

**Affiliations:** 1 Sydney School of Public Health, Faculty of Medicine and Health, The University of Sydney, Sydney, NSW Australia; 2 Wesfarmers Centre of Vaccines and Infectious Diseases, Telethon Kids Institute, Perth, WA Australia; 3 Department of General Practice, Western Sydney University, Campbelltown, NSW Australia; 4 School of Population Health, Curtin University, Perth, WA Australia; 5 Menzies Centre for Health Policy and Economics, University of Sydney, Sydney, NSW Australia; 6 Department of Gastroenterology and Hepatology, Royal Perth Hospital, Perth, WA Australia; 7 Curtin Medical School, Faculty of Health Sciences, Curtin University, Perth, WA Australia; 8 Burnet Institute, Melbourne, VIC Australia; 9 School of Public Health and Preventive Medicine, Monash University, Melbourne, VIC Australia; 10 Department of Infectious Diseases, Monash University, Clayton, VIC Australia; 11 Menzies School of Health Research, Charles Darwin University, Darwin, NT Australia; 12 Infectious Diseases Department, Royal Darwin Hospital, Darwin, NT Australia; 13 Department of Infectious Diseases and Microbiology, The Children’s Hospital at Westmead, Sydney, NSW Australia

**Keywords:** Randomised study, Dose–response, Bayesian design, Adaptive study, Direct-acting antiviral, Financial incentives, Hepatitis C, Primary care

## Abstract

**Background:**

Untreated hepatitis C virus (HCV) infection can result in cirrhosis and hepatocellular cancer. Direct-acting antiviral (DAA) therapies are highly effective and have few side effects compared to older interferon-based therapy. Despite the Australian government providing subsidised and unrestricted access to DAA therapy for chronic HCV infection, uptake has not been sufficient to meet the global target of eliminating HCV as a public health threat by 2030. This study will offer people with HCV financial incentives of varying values in order to evaluate its effect on initiation of DAA therapy in primary care.

**Methods:**

Australian adults (18 years or older) who self-report as having current untreated HCV infection can register to participate via an automated SMS-based system. Following self-screening for eligibility, registrants are offered a financial incentive of randomised value (AUD 0 to 1000) to initiate DAA therapy. Study treatment navigators contact registrants who have consented to be contacted, to complete eligibility assessment, outline the study procedures (including the requirement for participants to consult a primary care provider), obtain consent, and finalise enrolment. Enrolled participants receive their offered incentive on provision of evidence of DAA therapy initiation within 12 weeks of registration (primary endpoint). Balanced randomisation is used across the incentive range until the first analysis, after which response-adaptive randomisation will be used to update the assignment probabilities. For the primary analysis, a Bayesian 4-parameter EMAX model will be used to estimate the dose–response curve and contrast treatment initiation at each incentive value against the control arm (AUD 0). Specified secondary statistical and economic analyses will evaluate the effect of incentives on adherence to DAA therapy, virological response, and cost-effectiveness.

**Discussion:**

This project seeks to gain an understanding of the dose–response relationship between incentive value and DAA treatment initiation, while maximising the number of people treated for HCV within fixed budget and time constraints. In doing so, we hope to offer policy-relevant recommendation(s) for the use of financial incentives as a pragmatic, efficient, and cost-effective approach to achieving elimination of HCV from Australia.

**Trial registration:**

ANZCTR (anzctr.org.au), Identifier ACTRN12623000024640, Registered 11 January 2023 (https://anzctr.org.au/Trial/Registration/TrialReview.aspx?id=384923&isReview=true).

## Administrative information

Note: the numbers in curly brackets in this protocol refer to SPIRIT checklist item numbers. The order of the items has been modified to group similar items (see http://www.equator-network.org/reporting-guidelines/spirit-2013-statement-defining-standard-protocol-items-for-clinical-trials/)

**Table:** 

Title {1}	Randomised controlled dose–response study of financial incentives to motivate treatment for hepatitis C with direct acting antivirals among Australian adults (The **M**ethodical evaluation and **O**ptimisation of **T**argeted **I**ncenti**V**es for **A**ccessing **T**reatment of **E**arly-stage hepatitis **C**: MOTIVATE-C): Study protocol
Trial registration {2a and 2b}.	ANZCTR (anzctr.org.au) Identifier ACTRN12623000024640
Protocol version {3}	20 September 2023; Version 3
Funding {4}	2020 Medical Research Future Fund (MRFF)—PPHR Initiative—Efficient use of existing medicines (#2,007,164)
Author details {5a}	Parveen Fathima,^1,2^ parveen.fathima@sydney.edu.au Mark Jones,^1,2^ mark.jones1@sydney.edu.au Reena D’Souza,^2^ Reena.D’Souza@telethonkids.org.au James Totterdell,^1^ james.totterdell@sydney.edu.au Nada Andric,^1^ nada.andric@sydney.edu.au Penelope Abbott,^3^ P.Abbott@westernsydney.edu.au Richard Norman,^4^ richard.norman@curtin.edu.au Kirsten Howard,^5^ kirsten.howard@sydney.edu.au Wendy Cheng,^6,7^ Wendy.Cheng@health.wa.gov.au Alisa Pedrana,^8,9^ alisa.pedrana@burnet.edu.au Joseph S Doyle,^8,10^ joseph.doyle@burnet.edu.au Jane Davies,^11,12^ Jane.Davies@menzies.edu.au Thomas Snelling^1,13^ tom.snelling@sydney.edu.au 1. Sydney School of Public Health, Faculty of Medicine and Health, The University of Sydney, Sydney, NSW, Australia 2. Wesfarmers Centre of Vaccines and Infectious Diseases, Telethon Kids Institute, Perth, Western Australia, Australia 3. Department of General Practice, Western Sydney University, Campbelltown, New South Wales, Australia 4. School of Population Health, Curtin University, Perth, Western Australia, Australia 5. Menzies Centre for Health Policy and Economics, University of Sydney, Sydney, NSW, Australia 6. Department of Gastroenterology and Hepatology, Royal Perth Hospital, Perth, Western Australia, Australia 7. Curtin Medical School, Faculty of Health Sciences, Curtin University, Perth, Western Australia, Australia 8. Burnet Institute, Melbourne, Victoria, Australia 9. School of Public Health and Preventive Medicine, Monash University, Melbourne, Victoria, Australia 10. Department of Infectious Diseases, Monash University, Clayton, Victoria, Australia 11. Menzies School of Health Research, Charles Darwin University, Darwin, NT, Australia 12. Infectious Diseases Department, Royal Darwin Hospital, Darwin, NT, Australia 13. Department of Infectious Diseases and Microbiology, The Children's Hospital at Westmead, Sydney, NSW, Australia
Name and contact information for the trial sponsor {5b}	The University of Sydney Camperdown, NSW 2006.
Role of sponsor {5c}	The study sponsor and funder did not contribute to the study design and will not be involved in the conduct of the study or in the analyses of the results.

## Introduction

### Background and rationale {6a}

Hepatitis C virus (HCV) infection causes acute and chronic forms of hepatitis. In chronic HCV infection the disease may progress through worsening liver fibrosis to decompensated cirrhosis and/ or hepatocellular carcinoma and the need for liver transplantation [1]. HCV elimination is theoretically achievable thanks to the development of highly effective direct-acting antiviral (DAA) therapies for HCV infection. DAA therapies are associated with a viral cure rate of above 90% regardless of cirrhosis status, with significant improvement in liver function and reduced progression to hepatocellular cancer and end stage liver disease [2, 3]. Compared to older interferon-based therapy, DAA treatment is short (8–12 weeks vs 6–12 months) and has few side effects [4]. In March 2016, Australia became one of the first countries to make DAA therapy broadly accessible to adults with chronic HCV infection. Between March 2016 and December 2022, an estimated 100,684 people living with HCV infection initiated treatment [5]. However, after an initial surge in uptake of DAA therapy, treatment rates have fallen, and an estimated 74,400 Australians remained untreated at the end of 2022 [5].

Pragmatic strategies are needed to ensure people with early-stage chronic HCV infection access DAA therapy to prevent transmission and to avoid the debilitating and costly consequences of late-stage liver disease. Many people with HCV infection are socially marginalized and have difficulty accessing healthcare [5, 6]. Navigation by peers or other support workers has been shown to help people with complex health needs to access and adhere to care, including people with HCV [6].

Direct financial incentives have been shown to be beneficial for improving some desirable health-related behaviours, including vaccine uptake, cessation of smoking, and short-term treatment adherence [7, 8]. However, to date, few studies [9–12] have evaluated the use of financial incentives for short course HCV treatment and there is uncertainty regarding what value of incentives may be required to motivate initiation of DAA therapy. We postulate that a dose–response (incentive vs initiation of therapy) relationship exists and that characterising this relationship is needed to inform the incentivisation of HCV treatment.

The **M**ethodical evaluation and **O**ptimisation of **T**argeted **I**ncenti**V**es for **A**ccessing **T**reatment of **E**arly-stage hepatitis **C** (MOTIVATE-C) study is a novel dose-ranging study that aims to evaluate the effect of financial incentives on the propensity of people with chronic HCV infection to initiate DAA therapy in the context of a navigator-led patient support program.

### Objectives {7}

The primary aim of MOTIVATE-C is to implement, evaluate, and optimise the use of financial incentives to motivate initiation of DAA therapy in primary care settings among people with chronic HCV infection. Specific questions, in the context of a patient support program, are:

1. Does offering a financial incentive to people with HCV to initiate DAA therapy in primary care increase the probability of treatment initiation compared to usual care (no incentive)?
2. What is the dose–response relationship for the range of financial incentives offered and the probability of treatment initiation?
3. What participant incentive is optimal in terms of minimising the costs expended under the incentive program relative to the costs averted from the progression of untreated chronic HCV?

The secondary aim of MOTIVATE-C is to examine the extent to which primary care provider co-incentives modify the probability of treatment initiation. Specific research questions for this aim are:

1. Does offering primary care providers a co-incentive payment modify the probability of treatment initiation?
2. How is the dose–response relationship modified by a co-incentive offered to the primary care provider?
3. What combination of participant and primary care provider incentive is optimal in terms of minimising the costs expended under the incentive program relative to the costs averted from the progression of untreated chronic HCV infection?

### Trial design {8}

MOTIVATE-C is a pragmatic, factorial, dose–response study that includes sequential analyses and Bayesian adaptive design elements including stopping rules and response-adaptive randomisation. The design has a fixed ‘dose’ (incentive) range for participant incentives, from AUD 0 to AUD 1000 in AUD 50 increments with the AUD 0 dose used as the reference/control dose. For prescribers, the amount of the co-incentive is either AUD 0 or AUD 100.

At the time of registration for the study, participants are randomised to one of the participant incentive amounts. The randomisation weights are balanced across the available incentive amounts until the first interim analysis. While the zero dose retains a fixed allocation weight over the duration of the study, the remaining allocation weights are updated proportional to the updated probabilities that each arm (incentive amount) exceeds a minimal effective dose criterion and that the incentive amounts have a greater probability of response than lower value incentives. The minimum effective dose (MED) criterion is achieved if there is a 0.95 probability that a particular incentive amount results in a ten-percentage point increment (in absolute terms) above the response on the control arm (AUD 0). Futile doses (incentive amounts) are those that have a probability of < 0.2 of being minimally effective and will exit the randomisation which ceases further enrolment to these incentive amounts. If the probability of the MED criterion met is < 0.2 for all incentive amounts, then enrolment will be suspended to consider redesign, necessitating protocol and analysis plan updates.

The design involves regular analyses, which start once all participants enrolled in the first 6 months have reached 12 weeks post-registration, and then every 4 months or after the commitment of AUD100,000, whichever occurs sooner. These analyses will estimate the dose–response curve by fitting a four-parameter EMAX model and calculating the posterior probability of response for all arms. The study will continue until the total study funds available for incentives for participants, co-incentives for nominated primary care prescribers, and compensatory payments (described below) have been expended. A final analysis will be undertaken at the end of the study to address secondary objectives and undertake the economic analyses.

## Methods: participants, interventions, and outcomes

### Study setting {9}

Participants across urban and rural Australia self-register for the study via text messaging (SMS) interactions facilitated by the registration component of a bespoke MOTIVATE-C management system, which was developed by the Adaptive Health Intelligence IT team based at the University of Sydney. The study has no designated enrolment nor treatment sites, although the study is promoted nationally through posters and flyers in electronic and print media (see ‘Recruitment {15}’ below).

### Eligibility criteria {10}

To self-register, a person must be an Australian adult (aged 18 years or older) residing in Australia, who is registered for Medicare (Australia’s universal health insurance scheme), and who self-reports as having active HCV infection. A person is ineligible if they:

- Are already receiving DAA therapy for HCV infection or have received DAA therapy in the previous 6 months,
- Have previously enrolled in the project,
- Are unable/unwilling to provide informed consent or to complete follow-up, or
- Are unable to commence DAA treatment within 12 weeks of registration due to a contra-indication (e.g., pregnancy, or breastfeeding)

### Who will take informed consent? {26a}

Treatment navigators are project staff who have a dedicated role which involves one-on-one participant engagement to ensure participant understanding and commitment to the study. The treatment navigator contacts the participant to collect data and monitor their progress at key stages over the 12 weeks following registration.

Consent in MOTIVATE-C occurs in two stages. First stage consent (for registration) is implied when potential participants self-register via the initial SMS in which they report their self-assessed eligibility and desire to learn more from a project treatment navigator. Second stage consent occurs when the registrant is contacted by the treatment navigator who verbally explains what the project entails, including what participant data will be collected according to the ethics-approved participant information sheet (PIS). To proceed, the registrant must have their verbal consent documented by the navigator in the MOTIVATE-C study portal.

### Additional consent provisions for collection and use of participant data and biological specimens {26b}

N/A. There are no additional consent provisions for collection and use of participant data. No biological specimens are collected for the study.

## Interventions

### Explanation for the choice of comparators {6b}

Currently, there is little understanding about what constitutes an effective incentive value in this patient population. In consultation with stakeholders including people with lived experience of HCV infection and with injecting drug use, we have set a range from AUD 0 to 1000 in increments of AUD 50. The upper bound for the incentive values was selected based on stakeholder feedback as the value above which one would expect little incremental gain in the propensity to commence DAA treatment. The lower bound of AUD 0 provides a proxy for the status quo, i.e. absence of a financial incentive. The estimated response on each incentive value will be compared to the reference value (AUD 0) to determine whether it constitutes a minimally effective dose. However, it must be noted that all participants, including those assigned AUD 0 incentive, will have access to the navigator support program and will be eligible for a compensatory payment for HCV testing (see below), so any effect of the interventions need to be interpreted within that context.

### Intervention description {11a}

The intervention is a financial incentive for the participants delivered within the context of a patient support program (i.e. with support from treatment navigators); further details on the nature of the support are provided below. Registrants will be randomised to be eligible for an incentive, ranging in value from AUD 0 to 1000 (inclusive) in AUD 50 increments to be paid on evidence that the DAA medication has been dispensed (DAA treatment initiation) within 12 weeks of registration.

The incentive is offered either in conjunction with, or without, an offer of a co-incentive payment to the participant’s nominated primary care DAA prescriber. This prescriber is randomised (1:1) to a co-incentive payment of AUD 100 or no payment (AUD 0). If the participant does not nominate a preferred primary care prescriber, the participant is referred to a default study prescriber. Default prescribers are not randomised to a prescriber co-incentive payment.

Participants incentives’ and prescribers’ co-incentives are either paid into a digital debit card on a mobile device (smartphone) or else into a physical debit/gift card.

### Criteria for discontinuing or modifying allocated interventions {11b}

The study uses adaptive design features, including response-adaptive randomisation and early stopping rules for futility. The initial randomisation weights are equal across the available incentive values and will be adapted over time to remain proportional to the updated probabilities that (1) each arm exceeds a minimal effective dose criterion and that (2) the incentive amounts have a greater probability of response than lower value incentives.

The interim analyses will estimate the dose–response curve of the incentive value versus treatment initiation relationship by fitting a four-parameter EMAX model for calculating the posterior probability of response for all arms. From the third interim analysis onwards, a futility rule is activated to determine whether a minimum threshold response is present. In the case that the minimum threshold is not met, enrolment will likely be suspended, and alternative dose ranges considered. The study will continue until the total funds available for incentives, co-incentives, and compensatory payments have been expended. No other decision criteria have been pre-specified.

### Strategies to improve adherence to interventions {11c}

The participant is informed by the navigator at their first meeting (and in subsequent follow-up meetings) that, to be eligible for the incentive, evidence of DAA treatment initiation must be provided within 12 weeks of registration. Regardless of any financial incentives offered, the management of all participants is supported by a trained treatment navigator.

### Relevant concomitant care permitted or prohibited during the trial {11d}

All participants will receive support through a personal treatment navigator. Given Australia’s commitment to the World Health Organization’s viral hepatitis strategy for the global elimination of hepatitis C [13], it is possible that other strategies relating to HCV treatment will be available concomitant to the study. This may include use of peer and other support workers, and promotion of point-of-care testing for HCV. While we are not aware of use of financial incentives in Australia, it is conceivable that overlap between MOTIVATE-C and similar initiatives may occur during the lifespan of our study. Participants in other studies and those receiving other supports to assist initiating and/or continuing DAA therapy are not explicitly excluded.

### Provisions for post-trial care {30}

There are no provisions for participant management after completion of the study. Any ongoing management relating to HCV infection will be at the discretion of the participant’s nominated care provider.

### Outcomes {12}

#### Primary endpoint

Initiation of DAA therapy for HCV within 12 weeks of registration, as evidenced by the valid dispensed DAA medication.

#### Secondary endpoints

- Evidence of having had a HCV PCR test (regardless of result) within 12 weeks (84 days) of registration
- Number of scheduled/recommended days in which DAA therapy was not taken, irrespective of the prescribed duration of therapy (i.e. 8 weeks or 12 weeks):
  - ◦ < 7 days,
  - ◦ 7–13 days,
  - ◦ 14–20 days,
  - ◦ 21–27 days,
  - ◦ 28 + days,
  - as self-reported (via SMS or to the navigator) at approximately 28 days after the expected end date of therapy (DAA therapy usually has a duration of 2–3 months).
- Sustained virological response (SVR), defined as a negative HCV RNA PCR test at any time from at least 4 weeks (28 days) after completion of DAA therapy and before 12 months after registration.

### Participant timeline {13}

The main steps in the participant timeline are (see Table 1):

**Table 1:** Study-related events and the associated participant timeline

Events	Time point	Data collected	Type of contact
Registration and randomisation	On Registration – Time 0	Date of registration	SMS interface
Enrolment	As soon as practicable after registration, and not ≥ 12 weeks afterwards	Date of enrolment	Navigator phone/video call
PCR confirmation	As soon as practicable after enrolment, and not ≥ 12 weeks after registration	Evidence and date of HCV RNA PCR testing	Navigator phone/video call
Treatment initiation	As soon as practicable after PCR confirmation, and not ≥ 12 weeks after Registration	Evidence and date of dispensed DAA medication (**Primary outcome**)	Navigator phone/video call
Mid-treatment check-in	14 to 27 days after treatment initiation	Any adverse events (any untoward health-related events)	Navigator phone/video call
End-of-treatment check-in	4–5 weeks after scheduled treatment completion	Participant’s adherence to treatment (number of missed dosages)	SMS-based survey and/or Navigator phone/video call
SVR check	4 to 24 weeks after treatment completion	Evidence and date of SVR result	Navigator phone/video call

#### Registration

The SMS-based registration interface (underpinned by the Twilio platform—https://www.twilio.com), once initiated by the participant, prompts the user to respond to a series of self-assessed eligibility questions. Appropriate responses to all eligibility questions progress the potential participant through to successful registration, at which point they will be considered ‘registrants’ and will be informed of the randomly generated incentive amount they will be eligible for in return for providing evidence (within 12 weeks) of initiating DAA therapy. If the registrant confirms through the SMS registration interface that they wish to proceed with the project and are willing to be contacted by a treatment navigator, they are sent a link to the PIS on the project website and informed to expect contact from a treatment navigator within 72 h.

#### Enrolment (and consent)

The treatment navigator contacts (via telephone) all registrants who indicate their desire to be contacted. The navigator assesses and confirms the registrant’s eligibility for enrolment. After this, they explain the procedures, processes and data collection requirements of the project and clarify roles and responsibilities. The registrant becomes an ‘enrolled participant’ once they provide verbal consent (as detailed above) for participation in the study, and this date is defined as the date of enrolment.

#### Nomination of a preferred treatment prescriber

Following receipt of consent, participants are invited to nominate a primary care provider as their preferred DAA treatment prescriber. If the participant nominates a preferred prescriber, the navigator initiates the randomisation of the co-incentive payment for the prescriber via the navigator portal, another component of the MOTIVATE-C management system. The navigator then contacts (email, phone and/or fax) the participant’s preferred prescriber to provide a brief description of the project and to inform them that an un-named participant has nominated them as their preferred prescriber. If a co-incentive payment of AUD 100 has been assigned at randomisation, the preferred treatment prescriber will also be notified that they are eligible for a co-incentive payment on demonstration of treatment initiation by the participant.

If the participant does not nominate a preferred prescriber, or if their nominated prescriber indicates they are unwilling or unable to accept the participant, the navigator helps the participant to identify an alternative DAA treatment prescriber from a default list of experienced primary care HCV treatment providers. Default prescribers are not randomised to a co-incentive payment but are instead provided a compensation payment of AUD 50.

#### Evidence of HCV testing and dispensed DAA medication

At enrolment, the treatment navigator explains to the participant that it is expected that they will, as soon as practicable, make an appointment with the treatment prescriber (either face-to-face or via telehealth), receive a pathology referral to confirm active HCV infection by PCR (unless the prescriber deems it not necessary), and attend a pathology collection centre for confirmatory HCV testing. On receipt of the results of the confirmatory HCV PCR test(s), it is expected that the participant will inform the navigator (via video call or SMS) of the results (irrespective of whether positive or negative). Unless the HCV PCR test excludes active infection, the participant will need to obtain a prescription for DAA therapy from the prescriber, although the appropriateness of therapy and the decision to prescribe remains at the sole discretion of the prescriber in consultation with the participant; a prescriber also has the discretion to refer to a specialist prescriber if deemed clinically necessary. The participant is expected to fill the DAA prescription at any pharmacy.

Upon sighting (via video call or SMS) and validating the positive HCV test results and the dispensed DAA, the navigator initiates payment of the HCV test compensation (AUD 105) and the treatment incentive (if randomised to a non-zero amount) to the participant as either a digital or physical debit card. If applicable, a co-incentive payment for the nominated treatment prescriber (or compensation payment for a default prescriber) is also initiated as either a digital or physical debit card. If the HCV test results exclude active infection, the navigator initiates payment of the HCV test compensation (AUD 105) and documents the negative result on the navigator portal, and nothing further is required from the participant for the project. Where applicable, if the participant has not provided evidence of a filled DAA prescription within 4 weeks of testing, the navigator reminds the participant of the requirements to be eligible for an incentive payment.

#### Follow-up

Following confirmation that DAA has been dispensed, the participant is informed that they will be contacted in:

(i) 2–4 weeks following treatment initiation to confirm their receipt of the compensation + / − incentive payment and to document any untoward health-related events, and
(ii) 4–5 weeks after the expected date of completion of DAA therapy to facilitate a follow-up appointment with their treatment prescriber to test for a viral response (negative HCV PCR at least 28 days after completing therapy). At this end of treatment follow-up, the navigator also confirms and documents the participant’s self-reported adherence to treatment (recalled number of missed days of medication).

#### Evidence of SVR

The navigator organises a final follow-up call to sight and verify the participant’s end-of-therapy HCV PCR results, following which the participant receives AUD 75 as an HCV test compensation payment via digital or physical gift card. This payment is provided whether a viral response has been achieved or not, and this completes the study for the participant.

#### Discontinuation/withdrawal of participants

Enrolled participants may withdraw from the study at any time and consent may be reassessed by the navigator across the duration of the study to ensure it is accurate and up to date. Consent for any data collected to be used for future research purposes may also be invoked or withdrawn by the participant at any time.

### Sample size {14}

The intervention is the offer of a financial incentive at registration, which may subsequently become an actual payment upon evidence of initiating DAA therapy. Consequently, the sample size will be constrained by the realised dose–response curve and the fixed project budget awarded by the funding body. For example, if the dose–response moves from its lowest to highest response (treatment initiation) at relatively low value incentives, then the randomisation mechanism will lead to participants being preferentially allocated to lower value incentives allowing for more participants to be enrolled than when higher incentive values are required to motivate treatment initiation.

Through simulation over a range of scenarios, the expected total sample size ranged from 580 to 2000, dependent on the specific form of the dose response considered. In the null case, that is where there was no increase in the DAA initiation over the entire dose–response range, the study was suspended for futility in all simulated trials with an expected sample size of 580 enrolled participants. Across all scenarios, the type-I error was maintained below 6%. Non-null scenarios focused on a baseline response of approximately 15% and an increment of 20 percentage points (in absolute terms) across the incentive range. Depending on the shape of the dose–response curve, the probability of identifying an effective dose ranged from 80 to 99%.

### Recruitment {15}

To identify potential participants and to encourage registration, the project is broadly advertised through display of flyers, posters, and bulletins in electronic or print media. Ethics-approved promotional material state that people with HCV who participate may be eligible for a payment if they commence DAA therapy. Promotional materials include the URL and/or a QR code for the project website. Following engagement and discussions with the relevant stakeholders, the promotional material is displayed on public or electronic notice boards in primary care health services, community services, organisations providing needle and syringe programs, alcohol and drug services and prison re-integration programs, mobile point-of-care HCV testing sites, and at peer-based support organisations. Primary care clinicians are informed of the project via advertisements through professional networks and contacts, including primary health networks. Selective media buying may also be used to gain more public exposure to the project.

## Assignment of interventions: allocation

### Sequence generation {16a}

Participant randomisation uses an implementation of the mass weighted urn design [14] which produces a random sequence that targets an allocation ratio based on configurable weights. Equal allocation is used until the first analysis after which the allocation weights are updated based on the joint posterior from the primary analysis model. The weights are computed as:

$${w}_{k}\propto \sqrt{\frac{{\rho }_{k}{\phi }_{k}{\sigma }_{k}^{2}}{{n}_{k}+ 1}}$$

for $k>1$(the AUD 0 dose has fixed allocation throughout) and where:

- $k$ corresponds to the dose index (AUD 0 being 1, AUD 1000 being 21)
- ${\rho }_{k}$ corresponds to the probability of effectiveness (i.e. $\ge$ MED threshold of 0.95)
- ${\phi }_{k}$ corresponds to the first difference in the probability of response at, namely ${\phi }_{k}={p}_{k}-{p}_{k-1}$ where ${p}_{k}$ corresponds to the posterior probability of response under arm $k$
- ${n}_{k}$ is the number of enrolments reaching the primary endpoint for arm $k$

Once the weights are computed, they are normalised to unity. There are no further planned restriction or stratification with the participant randomisation.

Prescriber co-incentive randomisation is based on a pre-generated sequence using permuted blocks with a range of block sizes and with 1:1 allocation to a co-incentive and no co-incentive.

### Concealment mechanism {16b}

The allocation mechanism for participants is concealed from all parties as the sequence generation is dynamic and initiated by the participant at the time of registration. The allocation sequence for nominated preferred prescribers is pre-generated and held on a secure server accessible only by the study statistician and the software administrators of the MOTIVATE-C management system.

### Implementation {16c}

The randomisation and allocation processes are coordinated by the MOTIVATE-C management system, which is a microservices application developed by the Health and Clinical Analytics team at the University of Sydney. The system is hosted by University of Sydney with secure access, full audit trial and redundancy. Randomisation functionality is contained within a dedicated service which exposes a RESTful API that can be called by other services within the MOTIVATE-C management system. The mass weighted urn design of the randomisation service produces a random sequence that targets an allocation ratio based on configurable weights. The approach facilitates the implementation of response-adaptive randomisation without major divergence from the specified allocation ratio, which is a problem commonly experienced under response-adaptive randomisation when using simple random sampling.

Registrants initiate their own randomised allocation via the SMS interaction under the previously described response-adaptive allocation scheme. The registrant’s mobile phone number is used to confirm that they are a unique person for the project and not previously registered. If confirmed to be a new registrant, a computer-generated randomised incentive is assigned to the registrant, and they are notified of this by text message. The randomised incentive value is associated with the registrant’s phone number used for the registration. Therefore, if a registrant does not proceed to enrolment after being randomised and then tries to re-register using the phone number used previously for registration, the registrant will not be re-randomised but will be re-notified of their previously assigned incentive.

Prescriber co-incentive allocations are initiated by the navigator via the navigator portal, another component of the MOTIVATE-C management system, only if the participant nominates a preferred prescriber. Again, the implementation of the randomisation sequence is encapsulated within the randomisation microservice, but for prescriber co-incentives, the sequence is simply taken sequentially from pre-generated permuted block randomised sequences with 1:1 allocation to co-incentive or no co-incentive.

In both scenarios, the assigned treatment values are pushed to a dedicated REDCap database, which is used to retain all trial data.

## Assignment of interventions: blinding

### Who will be blinded {17a}

Participants are unblinded to the intervention as the primary research question is predicated on their knowledge of the assigned incentive value (dose). Similarly, nominated preferred prescribers are unblinded as the secondary research question is again predicated on the prescriber’s knowledge of the co-incentive value. While treatment navigators are not aware of the registrant’s assignment prior to making contact, in most cases they become unblinded due to the extent of their interaction with the participant. Prescribers may also become aware of the value of any incentive assigned to a participant they treat if the participant volunteers this information.

The study statistician needs to access participant level data for the purposes of conducting analyses and budget tracking and is therefore also unblinded. Other operational staff are not privy to the individual assigned incentive amounts but will be aware of the number of enrolled participants and aggregated summaries of the total budget expended on incentives and compensatory payments.

### Procedure for unblinding if needed {17b}

N/A. The study is unblinded.

## Data collection and management

### Plans for assessment and collection of outcomes {18a}

Only the minimum data necessary to achieve the study objectives are collected on participants (see Table 2). The rationale for this minimalism stems from our knowledge that many people HCV are socially marginalised and subject to stigmatisation, discrimination, legal issues, and other negative social and structural determinants of health. Accordingly, many people with HCV are wary of intervention programmes and are resistant to having their personal information collected. The study-relevant participant information will be recorded/documented by the navigator on the online navigator portal.

**Table 2:** Participant demographic and clinical variables collected for the study and the timepoint of data collection

Variable	Justification/comments	Timepoint
**Registrants and enrolled participants**		
Phone number		At registration
Date of registration in the study,* i.e. the date that the participant gets randomised and is advised of the assigned incentive*	To track compliance with project timelines	
Prior treatment/current treatment for HCV	To determine eligibility	At the initial meeting with the navigator
Do you have a Medicare card		
HCV positive PCR test in the last 4 weeks		
**Enrolled participants only**		
Name	For participant identification and validation of evidence of testing/prescription/SVR	At the initial meeting with the navigator
Date of birth	To permit adequate description of the study population and validation of evidence of testing/prescription/SVR	
Sex		
Mailing address	Only if participant requests for a hardcopy of the PIS and/or physical gift card to be delivered	
Date of enrolment in the study, *i.e. the date that the participant provides verbal consent to the navigator*	To track compliance with project timelines	At the initial or follow-up meeting (if participant require more time) with the navigator
Date and results of the HCV test	Secondary endpoint and to trigger compensation payment and to track compliance with project timelines	At the navigator-participant meeting to demonstrate evidence of HCV test
Name of the nominated treatment prescriber (including name of the practice)	To enable provision of the compensation payment	At the navigator-participant meeting to demonstrate evidence of DAA prescription
Date of treatment initiation	Primary endpoint and to trigger incentive payment and to track compliance with project timelines	
Adverse events (any untoward health-related events including medical attendance or hospitalisation)	For safety monitoring	At the navigator-participant meeting 2–4 weeks following initiation of treatment
Self-reported number of scheduled/recommended doses of DAA not taken grouped into:	Secondary endpoint	28 days following completion of treatment
• < 7 days,		
• 7–13 days,		
• 14–20 days,		
• 21–27 days,		
• 28 + days		
Date and results of the SVR test	Secondary endpoint and to trigger final compensation payment	4–24 weeks following completion of treatment

### Plans to promote participant retention and complete follow-up {18b}

The treatment navigators will foster a positive relationship with the participants by providing support, including facilitation of appointments if requested, sending reminders of timelines, clarifying any queries and helping to resolve issues that arise during the participant’s progression in the study. At each navigator-participant contact, the participant is informed/ reminded of the requirements for the next step of the study and the associated timeline. Following registration, at each expected, pre-determined point of contact, the treatment navigator attempts to contact the participant for a maximum of three attempts, at least 24 h apart over a 14-day window period.

Furthermore, regardless of any financial incentives offered, all participants are eligible to receive compensatory payments for testing to confirm HCV infection prior to initiation of DAA therapy (AUD 105), and to undergo a test of HCV viral clearance after completion of DAA therapy (AUD 75). Default prescribers, who are not randomised to a co-incentive, are eligible to receive a compensatory payment for each DAA prescription (AUD 50).

### Data management {19}

Data entry, data cleaning, and data management are coordinated by the Health and Clinical Analytics team at the University of Sydney. Data entry is performed by the treatment navigators following relevant training. Data is entered into the previously described project-specific electronic management system and stored in a REDCap platform hosted in a local server.

Documents will be retained and/or disposed per the sponsor’s Research Data Management Policy. In the case of closure of the project, the sponsor will retain an identical replica of the platform database for 15 years or longer, as is required by the approving regulatory authorities. All documentation will be archived until 15 years after the end of the project or publication, whichever is later. Any electronic data recorded on the platform database will also be archived according to the standard procedures of the Sponsor relating to archiving of electronic data.

### Confidentiality {27}

Individual participant data will be held securely by the University of Sydney coordinating centre, as required and permitted by law, and divulged only as necessary to authorised staff directly involved in the project. On all project-specific documents, the participant will be referred to by a unique project number and/or code as in the central database, not by name or other identifying information. Confidentiality agreements are in place between third-party vendors (the SMS-platform provider and the debit card providers) to ensure that personal information and information about the research project will not be disclosed to any unauthorised entity.

### Plans for collection, laboratory evaluation, and storage of biological specimens for genetic or molecular analysis in this trial/future use {33}

N/A. Although the results of routine tests are documented, no laboratory or biological specimens are collected as part of MOTIVATE-C.

## Statistical methods

### Statistical methods for primary and secondary outcomes {20a}

The main statistical objective of MOTIVATE-C is to characterise a dose–response curve where the ‘dose’ corresponds to the allocated incentive value and the ‘response’ corresponds to the propensity to initiate DAA therapy. We characterise superior incentive amounts relative to the notion of a minimum effective dose, which we define as any dose that increases the propensity to initiate DAA therapy by 10 percentage points, in absolute terms, relative to the response on the reference value (AUD 0). We refer to this as the minimum effectiveness criterion.

A Bayesian four-parameter EMAX model will be used to model the dose–response on the log-odds scale, which will be transformed via the inverse link to the probability scale to obtain the posterior probability to initiate DAA therapy at each dose. The EMAX model has been shown to provide good fit for analysing dose–response data and is a pragmatic choice that trades off the need for assumptions versus its flexibility to capture a wide range of dose–response profiles. Sequential analyses will commence once all participants enrolled in first 6 months have reached 12 weeks post-registration. Subsequent analyses are scheduled to occur every 4 months thereafter or after AUD 100,000 is committed as participant incentives, whichever occurs first. At each analysis, we will estimate the propensity to initiate DAA therapy at each incentive value. Inference will be made from Markov chain Monte Carlo (MCMC) draws from the joint posterior.

MOTIVATE-C permits design adaptations as the study progresses that relate to the posterior view of the dose–response curve. We define an effective dose as one which increases the probability of response by more than 10 percentage points in absolute terms relative to the zero-dose response with probability greater than 0.95. We define the minimum effective dose as the lowest incentive value that meets the definition of effectiveness. Any dose for which the probability of effectiveness is less than 0.2 is considered futile. A stopping rule is defined based on futility such that if all incentive values meet the futility definition, then the study will be suspended for review. The other adaptation relates to response-adaptive randomisation, as detailed earlier.

Methods for secondary analyses include logistic and cumulative logit models (assuming proportional odds). An economic analysis will be used to identify an optimal incentive via a cost-effective analysis taking a health services perspective. Cost-effectiveness measures will be summarised as incremental cost-effectiveness ratios and net monetary benefit [15].

All analyses will be undertaken in a Bayesian framework with standard diagnostics run on MCMC chains and using posterior predictive checks for goodness of fit. Additional models may be investigated (and reported as post hoc) as sensitivity and stability checks.

A complete statistical analysis plan will be published separately in accordance with suggested guidelines [16].

### Interim analyses {21b}

Analyses will be conducted at several points over the duration of MOTIVATE-C. Sequential analyses will commence once all participants enrolled in first 6 months have reached 12 weeks post-registration. Based on an expected enrolment rate of two per day and a linear ramp up to this rate over the first 6 months, this equates to approximately 170–200 participants being observed at the first analysis, distributed evenly over the available incentive values. If fewer than 100 participants are available for analysis after 6 months, then the first analysis will be deferred at monthly increments until a minimum of 100 participants have reached 12 weeks post-registration.

Subsequent analyses are scheduled to occur every 4 months thereafter or after AUD 100,000 is committed as participant incentives, whichever occurs first. This rule may be varied as the study approaches exhaustion of awarded funds to ensure all committed funds can be honoured.

The primary analysis will be performed at each analysis and the results will be used to compute the weights for the response-adaptive randomisation. At each analysis there will be some participants who have registered but who have not yet reached 12 weeks follow-up post-registration; these participants will not be included in the current analysis but will be included in subsequent ones. Following each analysis, the results will be used to recompute the weights for the response-adaptive randomisation.

### Methods for additional analyses (e.g. subgroup analyses) {20b}

N/A. No subgroup analyses are planned.

### Methods in analysis to handle protocol non-adherence and any statistical methods to handle missing data {20c}

Missingness is inherent in the study design. Registrants who are insufficiently motivated by the proposed incentive (if any) may choose not to be contacted by a navigator, and participants who fail to initiate therapy with DAA may not contact treatment navigators by 12 weeks post-registration. Navigators will make every reasonable effort to confirm the outcome status of enrolled participants. Failure to enrol or failure to ascertain evidence of successful commencement of DAA therapy by participants will be interpreted as a failure of the intervention.

### Plans to give access to the full protocol, participant-level data, and statistical code {31c}

The ethics-approved full study protocol is available from the authors upon request. De-identified participant level data can be made available after study completion with appropriate ethics approvals. Statistical code can be shared in certain approved cases, subject to the author(s) properly attributing any derived code used for future work.

## Oversight and monitoring

### Composition of the coordinating centre and trial steering committee {5d}

The project steering committee meets quarterly and comprises representatives from the investigator team, independent stakeholders, and community members. The coordinating centre operational team meets every week and comprises project staff involved in the day to day running of the study, including the CPI, project coordinator, software developers, and treatment navigators.

### Composition of the data monitoring committee, its role and reporting structure {21a}

Two independent statisticians, with expertise in clinical research methods and Bayesian statistics, provide independent statistical oversight and monitor adherence to the pre-specified analyses and project decision making processes (as applicable). Details of each analysis will be provided by the study statistician, in confidence, to the statistical monitors for approval.

### Adverse event reporting and harms {22}

It is not anticipated that there will be any significant risk to the safety of participants given the intervention is a payment to the participant (and possibly their nominated prescriber) on commencement of DAA therapy. Nevertheless, when the navigator contacts the participant 2–4 weeks (at the mid-treatment check-in) after initiation of therapy with DAA, all health-related events following treatment initiation will be elicited and documented in the study database. This will be reviewed by the delegated project personnel to assign the seriousness and likely causal relationship of the adverse event to the intervention. We will only report adverse events deemed to be likely causally related to the intervention and occurring within 6 months of participant registration.

### Frequency and plans for auditing trial conduct {23}

A data monitoring plan, based on a risk-based approach, specifies for the data to be regularly monitored for completeness and quality by a delegated member of the study team.

### Plans for communicating important protocol amendments to relevant parties (e.g. trial participants, ethical committees) {25}

All changes to the protocol will be submitted to the relevant ethics committee(s) for approval and documented as an amendment in the trial registry. The study coordinator will be responsible for communicating all approved amendments to the investigators, and to study participants as necessary.

### Dissemination plans {31a}

Study findings will be disseminated as reports (including lay summaries) to stakeholders, conference presentations, and peer-reviewed publications.

## Discussion

Despite the large residual burden of HCV disease, DAA treatment uptake in Australia has slowed. This project aims to treat as many people with HCV as possible while gaining an understanding of the dose–response relationship between the value of incentives and treatment initiation. In doing so, we hope to inform an approach for the pragmatic, efficient, and cost-effective elimination of HCV from Australia.

### Behavioural economics and nudge theory

Through workshops with target patient groups, GPs, and other stakeholders, we have used the following insights from behavioural economics [8] to guide the design of a financial incentive study for treatment initiation with DAA therapy:

- Limits of education: Educating patients and GPs, by itself, is unlikely to be a strong motivator. We anticipate that offering financial incentives of sufficient value to initiate treatment will improve the uptake of treatment where education alone has been insufficient.
- Choice overload: Offering a single, simple (cash) incentive for a specific target action (prescription of DAA by a primary care prescriber) within a set timeframe is expected to be effective than promoting a range of therapy options or incentives to choose from over an indefinite timeframe.
- Immediacy: Most people with early-stage HCV infection have few, if any, symptoms; concern about future potential morbidity and death is a weak intrinsic motivator. Incentives are expected to be more effective if awarded immediately upon DAA initiation rather than being contingent upon adherence or completion of therapy.
- Social ranking: Incentives of sufficient value is expected to act as a signal of the societal expectation of DAA therapy which could indirectly increase the proportion of primary care practitioners prescribing, and of people with HCV infection who are treated, independent of the direct effect of the incentive on those offered the incentive. Prescriber incentives are expected to be more likely to be acceptable and to be effective if framed as earned ‘compensation’ for incurred costs, rather than as a reward.
- Loss aversion: Burden, costs and side effects of DAA therapy are expected to ‘loom larger’ than the future benefits to health. Incentives are expected to work best if framed as a potential *loss* rather than a potential *gain*, i.e. as a ‘risk of *losing* a reward’ if therapy is not commenced in time, rather than as a reward *gained* if therapy is commenced.
- Goal gradients: DAA therapy entails several steps (confirm active HCV infection, prescribe, fill prescription, monitor, confirm viral clearance, etc.). Incentives are expected to work best if tied to the more proximal goal of a filling DAA prescription rather than the distant target of DAA adherence or treatment completion.
- Salience of reward: Incentives are expected to work best if explicitly tied to initiation of DAA therapy and paid directly to the participant and/or the prescriber, rather than being implemented as a tax benefit or lumped with salary, insurance reimbursements, or practice payments.

### Ethical considerations

The offer of financial incentives may be criticised as a subtle form of coercion, or as having the potential to undermine intrinsic motivation for positive health behaviour. In some cases, the offer of a financial incentive may affect a person’s judgement about the risk of potential harm, undermining a person’s autonomy [17]. However, people do not always act how they would like to, so an alternative perspective is that financial incentives enhance rather than restrict autonomy as they can help people align their actions with their preferences. Furthermore, despite concerns about the safety of providing undirected financial remuneration to people engaged in substance abuse, other studies have found that cash incentives did not have a significant effect on rates of new drug use or increase in risk of relapse [18, 19].

### Limitations

In an ideal study, the proposed intervention, i.e. the offer of incentives of various values, would be compared against no incentive in the context of usual practice. At present in Australia, a number of community-based organisations offer peer-based treatment support and navigation, although this is not available universally. In MOTIVATE-C, incentives are offered in the context of a program of treatment support by trained navigators, and participants receive compensation for adhering to study-related procedures (for HCV testing before and after treatment) regardless of whether they are assigned to any incentive. Therefore, any observed effectiveness of incentives must be interpreted within this context. While the question of the effectiveness of incentives outside of a treatment support program and without compensation payments will remain unanswered, it was deemed important on ethical grounds that all participants are compensated for their time and that all stand to derive some benefit from participation, even if not assigned to an incentive [20].

In conclusion, we anticipate that the offer of financial incentives will be shown to be a straightforward, readily implementable, and cost-effective strategy for curing the 74,400 Australians still living with HCV [5], and thereby help Australia to achieve HCV elimination.

## Trial status

This is the third version of the protocol (20 September 2023). Recruitment began on 15 May 2023 and is expected to finish on 30 June 2025. At the time of this submission, 186 participants have been randomised with 116 enrolled.

## Data Availability

Individual participant data will be held securely by the coordinating centre, as required and permitted by law, and divulged only as necessary to authorised/approved staff directly involved in the project. De-identified participant level data can be made available after study completion with appropriate ethics approvals.

## Abbreviations

CPI: Coordinating Principal Investigator
DAA: Direct-acting antivirals
HCV: Hepatitis C virus
PCR: Polymerase chain reaction
PIS: Participant Information Sheet
SMS: Short Messaging Service
